# Immunomodulatory R848-Loaded Anti-PD-L1-Conjugated Reduced Graphene Oxide Quantum Dots for Photothermal Immunotherapy of Glioblastoma

**DOI:** 10.3390/pharmaceutics16081064

**Published:** 2024-08-13

**Authors:** Yu-Jen Lu, Reesha Kakkadavath Vayalakkara, Banendu Sunder Dash, Shang-Hsiu Hu, Thejas Pandaraparambil Premji, Chun-Yuan Wu, Yang-Jin Shen, Jyh-Ping Chen

**Affiliations:** 1 Department of Neurosurgery, Chang Gung Memorial Hospital at Linkou, Kwei-San, Taoyuan 33305, Taiwan; luyj@cgmh.org.tw (Y.-J.L.); kvreesha@gmail.com (R.K.V.);; 2 College of Medicine, Chang Gung University, Kwei-San, Taoyuan 33302, Taiwan; 3 Department of Chemical and Materials and Materials Engineering, Chang Gung University, Kwei-San, Taoyuan 33302, Taiwan; banendusunder@gmail.com (B.S.D.);; 4 Department of Biomedical Engineering and Environmental Sciences, National Tsing Hua University, Hsinchu 300044, Taiwan; 5 Research Center for Food and Cosmetic Safety, College of Human Ecology, Chang Gung University of Science and Technology, Taoyuan 33302, Taiwan; 6 Department of Materials Engineering, Ming Chi University of Technology, Tai-Shan, New Taipei City 24301, Taiwan

**Keywords:** photothermal therapy, immunotherapy, glioblastoma, graphene oxide quantum dot, R848, anti-PD-L1

## Abstract

Glioblastoma multiforme (GBM) is the most severe form of brain cancer and presents unique challenges to developing novel treatments due to its immunosuppressive milieu where receptors like programmed death ligand 1 (PD-L1) are frequently elevated to prevent an effective anti-tumor immune response. To potentially shift the GBM environment from being immunosuppressive to immune-enhancing, we engineered a novel nanovehicle from reduced graphene oxide quantum dot (rGOQD), which are loaded with the immunomodulatory drug resiquimod (R848) and conjugated with an anti-PD-L1 antibody (aPD-L1). The immunomodulatory rGOQD/R8/aPDL1 nanoparticles can actively target the PD-L1 on the surface of ALTS1C1 murine glioblastoma cells and release R848 to enhance the T-cell-driven anti-tumor response. From in vitro experiments, the PD-L1-mediated intracellular uptake and the rGOQD-induced photothermal response after irradiation with near-infrared laser light led to the death of cancer cells and the release of damage-associated molecular patterns (DAMPs). The combinational effect of R848 and released DAMPs synergistically produces antigens to activate dendritic cells, which can prime T lymphocytes to infiltrate the tumor in vivo. As a result, T cells effectively target and attack the PD-L1-suppressed glioma cells and foster a robust photothermal therapy elicited anti-tumor immune response from a syngeneic mouse model of GBM with subcutaneously implanted ALTS1C1 cells.

## 1. Introduction

Glioblastoma multiforme (GBM) is the most aggressive and common primary brain tumor in adults, posing significant diagnostic and therapeutic challenges [1]. Its invasive growth, robust angiogenesis, and resistance to conventional treatments such as surgery, radiotherapy, and chemotherapy (e.g., temozolomide and bevacizumab) contribute to poor prognoses with a median survival of 12–15 months and a five-year survival rate below 5% [2]. The immunosuppressive tumor microenvironment of GBM, characterized by programmed death ligand-1 (PD-L1) overexpression, hinders effective T-cell-mediated anti-tumor responses and underlines the requirement for advanced immunotherapeutic strategies for treating GBM patients [3].

In most recent research, it is evident that the GBM microenvironment is characterized by a highly immune-privileged site with an immunosuppressive nature, which exerts disruption in T-cell signaling at a systemic level and acts as an immunologically “cold” entity [4,5,6]. In such instances, the immunosuppressive response of glioma cells has been linked to an overexpression of PD-L1 on the cell surface, which usually binds to the programmed cell death-1 (PD-1) receptors located on activated T-cell surface and leads to T-cell exhaustion and anergy [7]. Hence, the cancer cells are protected from T cells by a protective mechanism referred to as a “molecular shield”. As a therapeutic alternative, inhibiting these immune checkpoints through the induction of externally administered antagonists could potentially interrupt such an immune-suppressing pathway. This interruption has the potential to unleash and amplify preexisting anti-cancer immune responses in T cells, leading to the destruction of tumor tissue [8]. Immune checkpoint inhibitors have surfaced by blocking the PD-L1 on the glioma cell surface or PD-1 on the T-cell surface, allowing T cells to recognize and kill cancer cells [9]. Immunotherapy drugs such as Pembrolizumab, Nivolumab, Durvalumab, and Atezolizumab, which are monoclonal antibodies against PD-L1, have been developed as immune checkpoint inhibitors [10]. They demonstrate positive therapeutic outcomes in melanoma and non-small cell lung cancer [11,12]. Furthermore, many immunotherapeutic approaches for GBM treatment based on immune checkpoint inhibitors have demonstrated promising results in clinical trials [13].

However, cancer cell invasion and migration, tumor heterogeneity, and weakened therapeutic outcomes in cancer immunotherapy face challenges after single treatments [14]. Toward this, a combination of multiple immune boosting mechanisms has the potential to enhance the anti-tumor efficacy at different stages of cancer immune cycles [15]. These stages encompass antigen release, antigen presentation, immune cell priming and activation, immune cell trafficking and infiltration into tumors, and initiating cancer cell apoptosis [16]. Researchers have shown that the lack of antigens constitutes a primary factor contributing to tumor resistance to immunotherapy [17]. To improve the cross-presentation of tumor-associated antigens and to provide co-activation of adaptive and innate immunity, implementing immunogenic cell death (ICD) is crucial [18,19]. The ICD allows dying cells to release or expose molecules on their surface, which can function as either adjuvant or danger signals for the innate immune system [20]. These signals are called damage-associated molecular patterns (DAMPs) where some of them are secreted or released from the cells and others become enriched on the outer leaflet of the plasma membrane [21]. The signals can release tumor antigen and thus provoke dendritic cell (DC) maturation and T-lymphocyte infiltration, and these cascade events of ICD turn the immune-suppressive tumors into immuno-responsive tumors [22].

Nanoparticles are considered as a potential therapeutic approach for drug delivery to increase drug bioavailability and reduce drug adverse side effects. They can be decorated with antibodies or bioactive molecules to further improve the therapeutic index [23]. Nanoparticles-based combinational cancer therapies for immunotherapy have been reported lately by combining with radiotherapy [24], or photothermal therapy (PTT) [25,26]. The PTT can eliminate tumor cells by producing heat using nanoparticles as a photothermal agent, which can transform light energy to heat through photothermal effect and raise tumor temperature for ablation of tumor cells [27]. Furthermore, the temperature rise during PTT can initiate ICD by altering several cancer cell endogenous factors. The dying cancer cell can release adenosine triphosphate (ATP) and DAMPs such as calreticulin (CRT). This upregulated release of DAMPs from cancer cells can function as a source of antigen, thus producing antigen-presenting cells (APCs) and promoting DC-associated tumor immunotherapy [28,29]. Thus, by combining nanoparticle-mediated near-infrared (NIR) light-induced PTT with ICD, photothermal immunotherapy could be achieved [30,31].

Previously, liposome–conjugated ICG in combination with NIR irradiation could effectively induce an anti-glioma photo-immune response by promoting both apoptosis and necrosis of tumors by recruiting CD8^+^ T cells [32]. As one of the synthetic toll-like receptor (TLR) activators, imidazoquinoline R848 is an immunoadjuvant TLR7/8 agonist gaining attention in cancer immunotherapy [33,34]. Once interacting with the TLR7 on the cancer cell surface, this synthetic TLR activator can lead to the release of cytokines and chemokines such as tumor necrosis factor-alpha (TNF-α), interleukin-12 (IL-12), and IL-6, thus creating DC activation and specific tumor immune response [35]. Lu et al. incorporated R848 in polydopamine (PDA) nanoparticles as an immuno-adjuvant to serve both diagnostic (imaging) and therapeutic purposes [36]. The PDA nanoparticles demonstrated a NIR-inducible PTT, which further induced ICD-assisted immune cell activation. The combination of the R848 drug with ICD could successfully eradicate primary breast cancer tumors by triggering the immune response. In another work, 2D material reduced graphene oxide (rGO) was utilized for facile preparation of nanoparticles used in neoantigen-assisted vaccine therapy. The intracellular reactive oxygen species formation property and biodegradable characteristics of rGO provide lymph-node-targeted delivery of CpG (oligodeoxynucleotide, a TLR-9 agonist). By co-loading CpG and neoantigen peptide on PEGylated rGO, the nanoparticle could exert anti-tumor efficacy by a local immune response in MC-38 tumor-bearing mice [37]. Other than rGO, other 2D carbon-based nanomaterials have also gained attention in cancer therapy [38,39]. With excellent stability, biocompatibility, and NIR light absorption ability [40], these nanomaterials can serve as promising nanocarriers for drug delivery and act as efficient photothermal agents [41]. Their capacity to activate macrophages and release cytokines further underscores their suitability for photo-immunotherapy.

**Figure 1:**
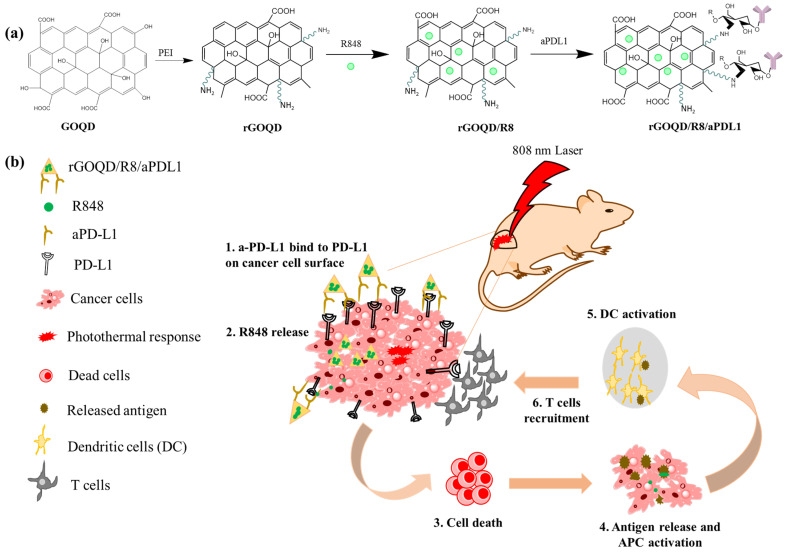
(**a**) Schematic illustration of the preparation of rGOQD/R8/aPDL1, including reduction of GOQD to rGOQD using polyethylene (PEI), immunoadjuvant drug R848 loading on rGOQD by π–π stacking (rGOQD/R8), and aPD-L1 conjugation on rGO/R8 using amine groups in rGOQD and aldehyde group in activated aPD-L1. (**b**) The photo-immunotherapy using rGOQD/R8/aPDL1 involves photothermal therapy and immune cell activation to exert an anti-tumor effect by (1) binding to the overexpressed PD-L1 receptors on tumor cell surface; (2) R484 release for activation of adaptive immune response; (3) photothermal-effect-induced cell death; (4) antigen release and antigen-presenting cells (APCs) activation; (5) dendritic cells (DCs) activation; (6) T cells recruitment.

Despite the emergence of numerous therapeutic targets and procedures, the most challenging type of brain cancer, GBM, still encountered failure in monotherapies. To address this challenge, the paradigm is shifting towards combination therapy focused on multiple ways of immune antigen production against glioma cells. Based on this combinatorial therapeutic strategy, the enhanced drug loading capacity, surface modification capabilities, precise aPD-L1 conjugation ability, and appreciable photothermal characteristics make reduced graphene oxide quantum dot (rGOQD) a good candidate for photo-immunotherapy [42]. Herein, we synthesize rGOQD from graphene oxide quantum dot (GOQD) by hydrothermal reduction process using polyethyleneimine as a reducing agent [43,44]. By utilizing amine functionalized rGOQD, we further surface conjugate anti-PD-L1 (aPD-L1) to rGOQD. On the other hand, a high drug loading capacity arising from the single-atom-thick honey-comb-arranged structure of rGOQD enables π–π stacking of resiquimod (R848) on its surface as an immunoadjuvant. This R848-loaded rGOQD was further surface conjugated with aPD-L1 to prepare intravenous injectable rGOQD/R8/aPDL1 nanoparticle, which can target the PD-L1 on the glioma cell surface for sustained release of the immunoadjuvant (Figure 1). When activated by NIR irradiation, the rGOQD can induce localized hyperthermia in tumors, promoting DC maturation and T-cell infiltration, thereby potentiating the immune response. Our combinatorial approach by integrating photothermal and immune-activating properties in a single nanoparticle presents a way for GBM treatment, which aims to overcome the current limitations of monotherapies and improve the therapeutic outcomes.

## 2. Materials and Methods

### 2.1. Materials

The graphene oxide quantum dot (GOQD, size = 20 nm) solution was obtained from CL Technology Co., Ltd. (New Taipei City, Taiwan). Resiquimod (R848) and branched polyethyleneimime (PEI) (average molecular weight = 800 kDa), Hoechst 33342, bovine serum albumin, and Live/Dead Cell Double-Staining Kit were obtained from Sigma-Aldrich (St. Louis, MO, USA). The anti-PD-L1 antibody (Durvalumab) was a gift from Chang Gung Memorial Hospital. The bicinchoninic acid (BCA) protein assay kit was obtained from Thermo Fisher Scientific (Waltham, MA, USA). For cell culture studies, Dulbecco’s modified Eagle’s Medium (DMEM), fetal bovine serum (FBS), and 0.25% trypsin-EDTA and penicillin-streptomycin were used. Phalloidin-iFluor 488 reagent for F-actin cytoskeleton staining, sulfo-cyanine 5.5 NHS ester (Cy5.5 NHS) for fluorescence labeling, and 3-(4,5-dimethylthiazol-2-yl)-5-(3-carboxymethoxyphenyl)-2-(4-sulfophenyl)-2*H*-tetrazolium (MTS) reagent for cell proliferation assays were purchased from Abcam (Cambridge, UK). A mouse astrocytoma cell line ALTS1C1 (BCRC 60582) and a mouse embryonic fibroblast cell line 3T3 (BCRC 60071) were obtained from Bioresource Collection and Research Center (Hsinchu, Taiwan).

### 2.2. Synthesis of Reduced Graphene Oxide Quantum Dots (rGOQD)

The rGOQD was synthesized via a modified hydrothermal method as previously described [44]. Initially, a GOQD solution (1 mg/mL) was ultrasonicated (amplitude 30, pulse on 30 s, pulse off 5 s, power 34 W) for 4 h to minimize nanoparticle aggregation and to reduce particle size. Subsequently, 10 mL of this GOQD solution was transferred to a three-necked flask. Branched PEI at 1% (*v*/*v*) concentration was then added dropwise under a nitrogen atmosphere. The mixture was heated to 100 °C with vigorous stirring for 3 h, during which a color transition from brown to black indicated the formation of rGOQD. After cooling to room temperature, the solution was dialyzed against deionized water (DIW) for 72 h using a 3 kDa molecular-weight-cut-off (MWCO) dialysis membrane to remove unreacted PEI. The purified rGOQD was stored at 4 °C for further use.

### 2.3. Preparation of R848-Loaded Anti-PD-L1-Conjugated Reduced Graphene Oxide Quantum Dots (rGOQD/R8/aPDL1)

The resiquimod (R848) was loaded onto rGOQD utilizing the π–π stacking interactions. A predetermined volume of R848 in dimethyl sulfoxide (DMSO) (200 μL) was incrementally introduced to the rGOQD solution (1 mg/mL). The mixture was subjected to rotational mixing at 4 °C in the dark for 24 h. After incubation, the nanoparticles were separated by centrifugation at 15,000× *g* for 15 min, and the rGOQD/R8 pellets were re-suspended in phosphate-buffered saline (PBS). Activation of aPD-L1 antibodies was achieved through the addition of 0.1 M sodium periodate (NaIO_4_) solution, administered dropwise to the antibody solution, followed by stirring for 20 min in the dark. This oxidation process facilitates the conversion of carbohydrate moieties in the antibodies to reactive aldehyde groups. The activated antibodies were then purified via a PD-10 desalting column by eluting with sodium carbonate buffer (0.2 M, pH 9.5). The purified antibodies were gradually introduced to rGOQD/R8 at a weight ratio of 1:6 and allowed to react for 5 min, where conjugation of activated antibody to the amine groups in the PEI moiety of rGOQD occurs through spontaneous covalent bond formation. To reinforce these covalent linkages, sodium borohydride (NaBH_4_) was added, followed by incubation at 4 °C for 1 h. The resultant rGOQD/R8/aPDL1 was collected by centrifugation at 15,000× *g* for 30 min, and the concentration of unconjugated antibodies in the supernatant was quantified using the Pierce™ BCA protein assay kit using a calibration curve established for aPD-L1.

### 2.4. Characterization of Nanoparticles

The morphology of synthesized nanoparticles was examined using a field emission transmission electron microscope (TEM) (JEM2100Plus, JEOL, Tokyo, Japan). The hydrodynamic size from dynamic light scattering (DLS) and zeta potential were determined using a Zetasizer Nano ZS90 (Malvern Panalytical, Malvern, UK) at 25 °C. Nanoparticle suspensions in PBS (50 µg/mL) were sonicated for homogeneity before measurement in a polystyrene cuvette. Absorption spectra for the nanoparticles and drugs were acquired using a UV/Vis spectrophotometer (GENESYS 150, Thermo Scientific). Fourier-transform infrared spectroscopy (FTIR) was conducted with a Tensor 27 FTIR spectrophotometer from Bruker (Ettlingen, Germany) for compositional verification of the GOQD, rGOQD, and rGOQD/R8/aPDL1 formulations. An UniDRON Raman spectrometer from UniNanoTech (Yongin, Republic of Korea), a D2 Phaser X-ray powder diffractometer from Bruker (Ettlingen, Germany), a X-ray photoelectron spectrometer from Thermo VG-Scientific (Waltham, MA, USA), and a Innova atomic force microscope from Bruker (Ettlingen, Germany) were used for Raman spectroscopy, X-ray diffraction (XRD), X-ray photoelectron spectroscopy (XPS), and atomic force microscopy (AFM) analysis, respectively.

### 2.5. Drug Loading and Release

The amount of R848 adsorbed to rGOQD was determined by measuring the absorbance at 320 nm. After incubating rGOQD and R848 in pH 7.4 PBS for 24 h, the supernatant was separated from R848-loaded rGOQD by centrifugation and the solution absorbance was determined via UV/Vis spectroscopy to quantify the concentration of unbound R848 with a linear calibration curve constructed for R848. The release profile of R848 from rGOQD/R8/aPDL1 was investigated at pH 5 and 7.4 to understand the impact of environmental pH on drug release kinetics. This drug release was conducted at room temperature using a dynamic dialysis technique with PBS. The R848 release from the nanoparticles was monitored at selected time intervals by measuring the UV absorbance at 320 nm.

### 2.6. In Vitro Photothermal Response

The photothermal capabilities of GOQD, rGOQD/R8, and rGOQD/R8/aPDL1 (100 µg/mL concentration) were assessed using an 808 nm NIR laser at a 1.5 W/cm^2^ power density. For the experiment, 1 mL aliquots of the nanoparticle suspensions were placed into Eppendorf tubes and exposed to the laser for 5 min. The temperature changes in the suspensions were recorded at 1 min intervals using a Teledyne FLIR thermal camera to visualize the temperature increase from the real-time thermal images. Deionized water (DIW) was used as a control to benchmark the temperature changes against those observed in the nanoparticle suspensions. Furthermore, the dose-dependent photothermal response of rGOQD/R8/aPDL1 was examined from 25 to 100 µg/mL concentrations.

### 2.7. Biocompatibility of Nanoparticles

The biocompatibility of the nanoparticles was assessed via the MTS assay to determine their potential toxicity toward 3T3 fibroblasts and ALTS1C1 cancer cells. Initially, 1 × 10^3^ cells per well were plated in a 96-well plate with 200 µL of DMEM and incubated for 24 h. Following this, the cells were exposed to various concentrations (25–100 µg/mL) of rGOQD/R8/aPDL1 for another 24, 48, or 72 h. Post incubation, the culture medium was discarded, and the cells were washed with PBS. Subsequently, the diluted MTS reagent in cell culture medium was added to each well and incubated for 3 h, after which the formation of formazan crystals was quantified by measuring the absorbance at 490 nm.

### 2.8. Intracellular Uptake of Nanoparticles

For intracellular uptake study, nanoparticles were first tagged with Cy5.5-NHS dye (10 µg/mL prepared in DMSO) for 24 h and purified by centrifugation. The intracellular uptake of rGOQD/R8 and rGOQD/R8/aPDL1 was determined quantitatively by seeding 1 × 10^6^ ALTS1C1 cells/well in 6-well plates, followed by 24 h incubation at 37 °C. After washing with PBS, cells were treated with Cy5.5-tagged rGOQD/R8 and rGOQD/R8/aPDL1 in DMEM for 4 or 24 h. After PBS washes, cells were trypsinized and re-suspended in flow tubes and the internalization of Cy5.5 dye was quantified using a flow cytometer.

### 2.9. Cytotoxicity of Nanoparticles with NIR Laser Irradiation

ALTS1C1 glioma cells were used to determine the cytotoxicity of R848-loaded rGOQD nanoparticles combined with NIR laser irradiation (rGOQD/R8+L and rGOQD/R8/aPDL1+L). Cells (5 × 10^3^ per well) were seeded in 96-well plates and incubated overnight at 37 °C with 5% CO_2_. After replacing the cell culture medium with various concentrations of nanoparticles prepared in the medium, cells were further incubated for 24 h and subsequently exposed to a 1.5 W/cm^2^ NIR laser for 5 min and the cell viability was determined via the MTS assay. The cytotoxicity was further verified using a cell double-staining kit by growing ALTS1C1 cells (1 × 10^5^ per well) in 6-well plates. After incubation with different nanoparticles, the cells were exposed to NIR laser irradiation and stained with Calcein-AM and propidium iodide (PI). The stained cells were imaged using a Nikon Eclipse Ti-U inverted microscope with appropriate fluorescence filters. The control group is cells cultured in a cell culture medium without nanoparticles.

### 2.10. Analysis of Antigen Presentation after Nanoparticle-Induced Photothermal Treatment

To evaluate the potential of antigen-mediated immune therapy after photothermal treatment, ALTS1C1 cells were cultured in confocal dishes at a density of 1 × 10^5^ cells/well. The cells were treated with rGOQD/R8 and rGOQD/R8/aPDL1 at 100 µg/mL concentration while the control group was treated with PBS. Following a 24 h incubation period, the cells were rinsed with PBS and exposed to an 808 nm laser for 5 min. Post irradiation, cells were fixed with 4% formaldehyde for 15 min, then permeabilized with 0.1% Triton X-100 for 20 min at 37 °C. After blocking with 1% BSA for 30 min at room temperature, cells were washed with PBS and incubated overnight at 4 °C with an anti-calreticulin antibody (rabbit monoclonal, Abcam, ab92516). Following PBS wash, cells were further incubated with goat anti-rabbit IgG H&L Alexa Fluor 488-conjugated secondary antibody for 1 h at room temperature in the dark. Finally, cells were stained with Hoechst 33348 for 15 min. The stained cells were visualized using a confocal laser scanning microscope (LSM-800, Zeiss, Oberkochen, Germany). The mean fluorescence intensity was quantified using PAX-it software (https://www.paxit.com) for comparison of the antigen presentation after photothermal treatment.

### 2.11. In Vivo Biodistribution

The biodistribution study was conducted following the approved guidelines of the Chang Gung University Institutional Animal Care and Use Committee. Six-week-old female C57BL/6J mice were utilized for these experiments. Tumor cells (ALTS1C1, 1 × 10^6^ cells in 200 μL PBS) were implanted subcutaneously into the left flank of the mice. Tumor volumes were determined using Vernier calipers and calculated using the formula v = L × W^2^/2, where L and W denote the longest and shortest dimensions of the tumor, respectively. Once the tumors reached 150 mm^3^, 100 μL Cy5.5-labeled rGOQD/R8 or rGOQD/R8/aPDL1 were intravenously administered (rGOQD dose = 10 mg/kg). In vivo imaging was performed 4 h post injection using a non-invasive in vivo imaging system (Xenogen IVIS 200, Caliper Life Sciences, Hopkinton, MA, USA). Major organs, including the heart, lungs, liver, spleen, kidneys, and tumor, were harvested for ex vivo fluorescence imaging to quantify and compare the nanoparticle accumulation in each organ and the tumor-targeting ability. The relative fluorescence intensity from each organ was normalized to the cumulative fluorescence from all examined organs and expressed as a percentage for comparing the biodistribution profile of the nanoparticles.

### 2.12. In Vivo Photothermal Effects

Following the intravenous injection of nanoparticles into the tail vein of mice with tumors measuring 150 mm^3^, the tumor area was subjected to a 5 min exposure of 808 nm NIR laser at a power density of 1.5 W/cm^2^. The rise in temperature due to the photothermal effect was recorded in real time using an infrared camera (Teledyne FLIR LLC, Wilsonville, OR, USA) throughout the duration of the NIR laser irradiation to visually document the temperature increments induced by the photothermal conversion ability of the nanoparticles.

### 2.13. In Vivo Anti-Tumor Efficacy

The in vivo anti-tumor activity was assessed using six-week-old female C57BL/6J mice obtained from the National Laboratory Animal Center, Taiwan. All procedures were conducted per the guidelines approved by the Institutional Animal Care and Use Committee (IACUC) of Chang Gung Memorial Hospital, Linkou, Taiwan. ALTS1C1 glioblastoma cells (1 × 10^6^ cells in 200 µL PBS) were subcutaneously implanted into the left flank of the mice. After the tumor size reached a volume of 60–100 mm^3^, the mice were randomly grouped into four groups, i.e., PBS (control), rGOQD/R8, rGOQD/R8+L, and rGOQD/R8/aPDL1+L. On day 10 post implantation, the mice received an intravenous injection of 200 µL of the nanoparticle solution. The tumors were subjected to a 5 min NIR laser irradiation (808 nm, 1.5 W/cm^2^) in the rGOQD/R8+L and rGOQD/R8/aPDL1+L groups. Subsequently, 200 µL nanoparticle administrations via tail vein injection were conducted every three days and NIR laser irradiation was used in the rGOQD/R8+L and rGOQD/R8/aPDL1+L groups as before. The therapeutic outcome was monitored every other day by measuring tumor volume and recording the body weight of each mouse. On day 19, a subset of mice from each group was sacrificed and the blood, major organs, tumors, and lymph nodes were harvested for subsequent analysis. Mice were humanely euthanized if the tumor volume exceeded 1000 mm^3^, and the survival duration of the remaining mice was recorded to construct the survival curve.

### 2.14. Histological Evaluation

After sacrificing the mouse, vital organs such as the hearts, lungs, livers, spleens, kidneys, and various lymph nodes (cervical, axillary, and inguinal) were harvested and fixed in 4% formaldehyde for subsequent histological analysis. The tissues, including the extracted tumor samples, were fixed in phosphate-buffered formalin, embedded in paraffin, and sectioned to a thickness of 5 μm for hematoxylin and eosin (H&E) staining. The sections were then processed for immunohistochemical (IHC) staining or immunofluorescence staining, utilizing primary antibodies against Ki-67 (Affinity Biosciences, Cincinnati, OH, USA), PD-L1 (Abcam), TNF-α (Affinity Bioscience), CD11c (Invitrogen, Waltham, MA, USA), CD86 (Invitrogen), CD4 (Sigma-Aldrich), and CD8a (Sigma-Aldrich). After overnight incubation with primary antibodies at 4 °C, sections were exposed to secondary antibodies conjugated with Alexa Fluor 488 or Texas Red for immunofluorescence staining or horseradish peroxidase (HRP)-conjugated secondary antibody for IHC staining. The slides were visualized using a confocal microscope or a Tissue FAXS inverted bright field scanning system (Tissue Gnostics GmbH, Vienna, Australia). Quantitative analysis was performed using the ImageJ software (version 1.54b).

### 2.15. Statistical Analysis

The data are presented as mean ± standard deviation (SD) and statistically analyzed by one-way analysis of variance (ANOVA) using the LSD test with significant difference at *p* < 0.05.

## 3. Results and Discussion

### 3.1. Synthesis and Characterization of Nanoparticles

**Figure 2:**
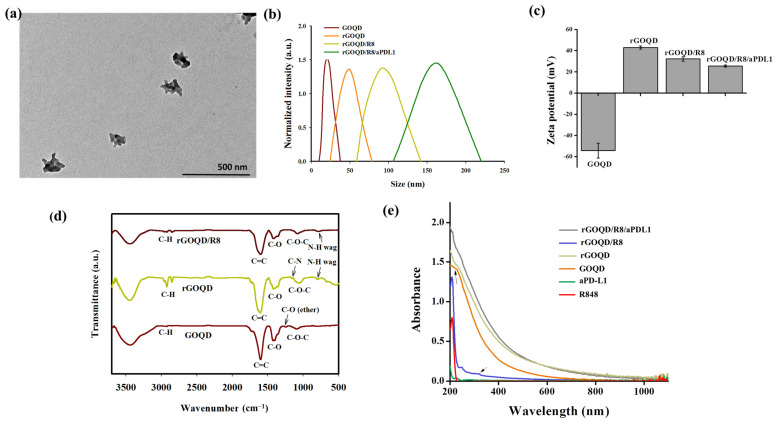
(**a**) The TEM image of rGOQD/R8/aPDL1 (scale bar = 500 nm). (**b**) The size distribution from dynamic light scattering analysis of GOQD, rGOQD, rGOQD/R8, and rGOQD/R8/aPDL1. (**c**) The zeta potential values of different nanoparticles (mean ± SD, *n* = 3). (**d**) The FTIR spectra of GOQD, rGOQD, and rGOQD/R8. (**e**) The UV-Vis spectroscopy analysis of GOQD, rGOQD, rGOQD/R8, and rGOQD/R8/aPDL1.

The GOQD with an average size of ~20 nm was utilized in a hydrothermal reduction process to synthesize the rGOQD. This process involved the use of polyethyleneimine (PEI) to facilitate the reduction of oxygenated functional groups in GOQD [43]. The TEM analysis revealed that the size of the resulting rGOQD is ~80 nm in diameter (Figure S1, Supplementary Materials). Following synthesis, rGOQD was loaded with the immunomodulatory drug R848 through π–π stacking interactions [38], and anti-PD-L1 monoclonal antibody was then conjugated to the rGOQD/R8 to improve their targeting capability and anti-tumor efficacy [45]. The size of rGOQD/R8/aPDL1 is still below 200 nm from TEM analysis but shows a rough contour when compared with rGOQD (Figure 2a). The DLS analysis supports the TEM analysis with monodispersed curves (Figure 2b) and PDI values below 0.3 to confirm the uniformity of the nanoparticles (Table 1). The average size of rGOQD increases over that of GOQD due to the hydration of the PEI chains in an aqueous environment, and R848 loading and aPD-L1 conjugation sequentially increase the particle size (Table 1). The zeta potential measurements validated the successful synthesis and functionalization of the nanoparticles (Figure 2c). Initially, the GOQD displayed a highly negative zeta potential (−54.2 mV), which, after reduction with PEI, became positive (43.2 mV). Loading with R848 shifts the zeta potential to 32.3 mV, indicating successful drug loading. The conjugation of aPD-L1 was confirmed as the zeta potential changes to 25.5 mV, which occurs by consuming the positively charged amine groups during the conjugation reaction (Table 1). This provides a stable covalent bond formation between the antibody and the nanoparticles [45]. The efficiency of antibody conjugation was 66%, with a nanoparticle-to-antibody ratio of 6:1, from BCA protein assays.

The FTIR spectroscopy confirms the chemical structure of the nanoparticles (Figure 2d). A broad peak within 3200–3500 cm^−1^ in the spectrum of GOQD corresponds to a high density of -COOH and -OH groups. The C–O–C, C–O and C=C stretching vibrations in GOQD are shown at 1095 cm^−1^, 1417 cm^−1^, and 1602 cm^−1^, respectively [46]. Compared with GOQD which shows an ether C–O stretching peak at 1246 cm^−1^, disappearance of this peak in rGOQD as well as appearance of a new peak at 1169 cm^−1^ from C–N stretching (primary amine) indicates successful reduction of GOQD by PEI to form rGOQD [43]. Also, the appearance of new peaks at 2932 cm^−1^ and 2852 cm^−1^ in rGOQD corresponded to the symmetric and asymmetric stretching of C-H in PEI to support its incorporation in rGOQD [47]. Furthermore, presence of an N-H wagging peak at 788 cm^−1^ in both the rGOQD and rGOQD/R8 spectra confirmed the PEI-mediated reduction of GOQD for antibody conjugation. The UV-Vis spectroscopy provided further confirmation of the nanoparticle composition. The characteristic peak for GOQD at 230 nm shifted to 263 nm in rGOQD, indicating the restoration of π-electron conjugation (Figure 2e). The peak at 320 nm from R848 appears in rGOQD/R8, affirming the successful loading of the drug within the nanoparticles (Figure S2, Supplementary Materials). The Raman spectroscopy, XRD, XPS, and AFM analysis of rGOQD/R8 further support its successful synthesis (Figure S3, Supplementary Materials).

**Figure 3:**
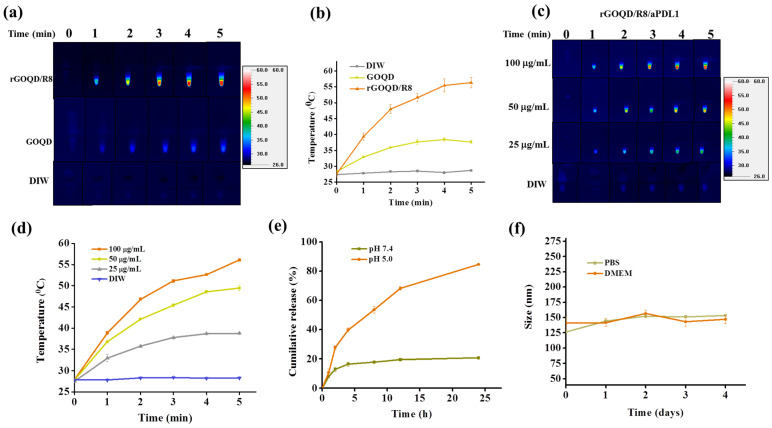
The photothermal images (**a**), and the corresponding temperature profiles (**b**) by irradiating GOQD or rGOQD/R8 (100 μg/mL) with 808 nm laser (1.5 W/cm^2^) for 5 min. The control is deionized water (DIW). The thermal images (**c**), and the corresponding temperature profiles (**d**) by irradiating 25–100 μg/mL rGOQD/R8/aPDL1 with 808 nm laser (1.5 W/cm^2^) for 5 min. (**e**) The in vitro release of R848 from rGOQD/R8/aPDL1 at pH 5 and 7.4. (**f**) The stability of rGOQD/R8/aPDL1 in PBS and DMEM cell culture medium by measuring the particle size from DLS. All data are represented as mean ± SD (*n* = 3).

The aqueous dispersions of rGOQD/R8 and GOQD were subjected to 808 nm NIR laser irradiation at a power density of 1.5 W/cm^2^ to assess their photothermal conversion capabilities. The results, illustrated in Figure 3a,b, indicate that rGOQD/R8 exhibits a significant temperature increase to 56 °C following 5 min exposure, while GOQD shows a moderate temperature elevation to 38 °C under identical conditions. The temperature increase rate is higher for rGOQD than that of GOQD due to the restored sp2 structure in rGOQD [48]. The enhanced photothermal effect of rGOQD/R8 suggests their potential application as efficient photothermal agents for cancer therapy [49]. The photo stability of rGOQD/R8 was confirmed from three consecutive on/off laser cycles (Figure S4a, Supplementary Materials). The effects of varying NIR power intensity on the thermal characteristics of the formulations was also investigated and higher laser power intensity can lead to faster temperature increases as well as higher final temperatures (Figure S4b, Supplementary Materials) [50]. The temperature rise induced by rGOQD/R8/aPDL1 is concentration-dependent (Figure 3c,d). A peak temperature of 56 °C was achieved at 100 μg/mL nanoparticle concentration and closely matches that of rGOQD/R8. These findings indicate that antibody conjugation does not affect the photothermal response of the nanoparticles as expected. Moreover, the ability to modulate the therapeutic heat by varying nanoparticle concentration provides a mechanism for tailoring the photothermal effect to the therapeutic requirements [50]. Control experiments involving deionized water (DIW) with identical laser irradiation conditions did not result in any notable temperature increase, confirming the photothermal response solely to the nanoparticles.

For the loading of resiquimod (R848) to rGOQD, 1 mg of rGOQD was mixed with a varying amount of R848. A standard curve of R848 was used to determine the unbound R848 in the solution and the drug loading content (the percentage of the amount of R848 loaded to the total amount of rGOQD applied in formulation) and loading efficiency (the percentage of the amount of R848 loaded to the total amount of R848 applied in formulation) was calculated (Figure S5, Supplementary Materials). By choosing 0.8 mg R848 as the best formulation, the drug loading content is 45.1 ± 5.0% and the drug loading efficiency was 67.0 ± 0.1%. This formulation was used for the release experiments. The release profile of resiquimod (R848) from rGOQD/R8/aPDL1 was evaluated under different pH values to simulate physiological (pH 7.4) and endosomal environments (pH 5). As depicted in Figure 3e, under physiological pH value, the drug demonstrated a controlled release behavior with less than 20% of the drug being released in 24 h. This indicates a strong π–π interaction between R848 and rGOQD, limiting premature drug leakage in physiological conditions. Conversely, the release profile at pH 5, akin to the endosomal microenvironment, showed that R848 in rGOQD/R8/aPDL1 released approximately 67% of the initial value in 12 h and reached a release plateau of 80% after 24 h. The noticeable pH-responsive drug release behavior suggests the accelerated release of R848 in acidic conditions, with which rGOQD/R8/aPDL1 can release this immunadjuvant after endocytosis or in an acidic tumor microenvironment [51].

The colloidal stability of rGOQD/R8/aPDL1 was tested in the cell culture medium (DMEM) and PBS (pH 7.4). Over a period of 4 days, the DLS analysis indicated negligible particle size change, endorsing excellent colloidal stability (Figure 3f). Furthermore, gross observation of the appearance of the suspension corroborated the DLS data, with no observable aggregation in both media over the test duration (Figure S6, Supplementary Materials).

### 3.2. In Vitro Studies

**Figure 4:**
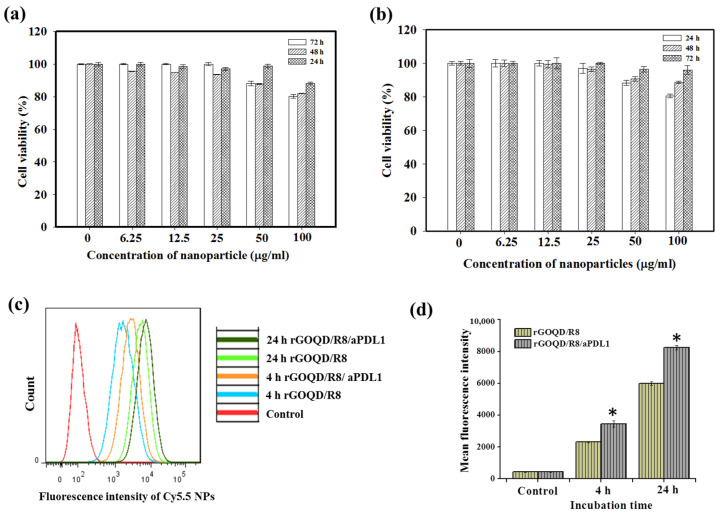
The biocompatibility of rGOQD/R8/aPDL1 was tested with 3T3 mouse embryonic fibroblast cells (**a**) and ALTS1C1 mouse glioma cells (**b**) with MTS assays at 24, 48, and 72 h. (**c**) The flow cytometry analysis of intracellular uptake of Cy5.5-tagged rGOQD/R8 and rGOQD/R8/aPDL1 after incubation with ALTS1C1 cells for 4 and 24 h. (**d**) The corresponding quantified fluorescence intensity from flow cytometry analysis of intracellular uptake of Cy5.5-tagged nanoparticles. * *p* < 0.05 compared with rGOQD/R8. All data are represented as mean ± SD (*n* = 3).

The biocompatibility of rGOQD/R8/aPDL1 nanoparticles is a pivotal factor before evaluating their therapeutic efficacy in vitro and in vivo. We performed an MTS cell viability assay to assess this parameter. Nanoparticles at varying concentrations (from 6.25 to 100 μg/mL) were incubated with 3T3 fibroblasts or the ALTS1C1 glioblastoma cells. As shown in Figure 4a,b, without laser irradiation, the rGOQD/R8/aPDL1 exhibited a non-toxic nature for both cell lines without eliciting significant cytotoxicity (>80% cell viability) across the nanoparticle concentration tested, endorsing its safe use for cancer therapy.

The intracellular uptake of Cy5.5-labeled rGOQD/R8 (non-targeted) and rGOQD/R8/aPDL1 (targeted) was qualitatively assessed by incubation with ALTS1C1 cells and intracellular Cy5.5 fluorescence intensity associated with internalized nanoparticles were measured using flow cytometry. The time-dependent fluorescence intensity increase within cells indicates successful intracellular uptake over a 24 h period, and fluorescence intensity distribution curve shifts to the right for rGOQD/R8/aPDL1 when compared with rGOQD/R8 at each time point (Figure 4c). Further quantitative analysis of mean fluorescence intensity from flow cytometry analysis demonstrated a significant increase in intracellular fluorescence signal intensity for rGOQD/R8/aPDL1 in comparison to rGOQD/R8 at both time points (Figure 4d). Overall, the surface-conjugated aPD-L1 will not only act as an antagonist but also enhance tumor targeting and intracellular uptake, potentially increasing therapeutic accumulation within tumor tissues [52]. The antibody-conjugated nanoparticle (rGOQD/R8/aPDL1) exhibits faster internalization kinetics and a higher internalization rate than the pristine rGOQD/R8 nanoparticles. This suggests that rGOQD/R8/aPDL1 can be used for robust recognition of glioma cells to deliver its R848 cargo from the targeting ability of the fragment antigen binding (Fab) region of aPD-L1, which was not disrupted during conjugation to rGOQD for recognizing the overexpressed PD-L1 on the cancer cell surface [53].

**Figure 5:**
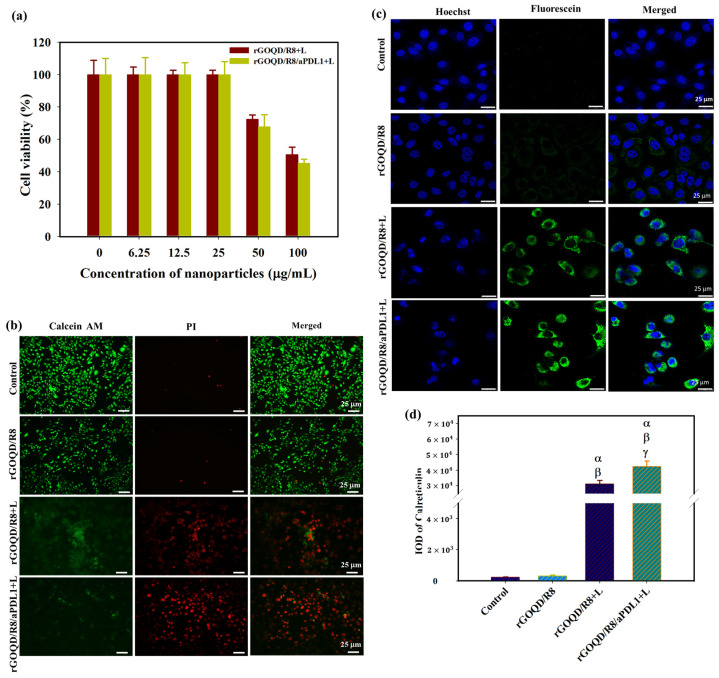
(**a**) The cell viability of ALTS1C1 cells after incubating cells with rGOQD/R8 (rGOQD/R8+L) or rGOQD/R8/aPDL1 (rGOQD/R8/aPDL1+L) at varying concentrations and irradiated with 808 nm laser at 1.5 W/cm^2^ for 5 min. (**b**) The fluorescence microscopy images of Calcein-AM and PI co-stained cells after incubation with different nanoparticles and 808 nm NIR treatment at 1.5 W/cm^2^ for 5 min. Cells treated with PBS and rGOQD/R8 without laser irradiation were used as controls. (**c**) The CLSM images of ALTS1C1 cells by staining with fluorescein-tagged anti-calreticulin antibody after different treatments. The rGOQD/R8+L and rGOQD/R8/aPDL1+L groups are irradiated with 808 nm NIR laser at 1.5 W/cm^2^ for 5 min. (**d**) The corresponding quantification of calreticulin fluorescence intensity by using the PAX-it software. All data are represented as mean ± SD (*n* = 3). ^α^ *p* < 0.05 compared to PBS, ^β^ *p* < 0.05 compared to rGOQD/R8, ^γ^ *p* < 0.05 compared to rGOQD/R8+L.

The photothermal therapeutic effect was assessed following exposure of nanoparticle-treated cells to an 808 nm NIR laser. As presented in Figure 5a, after applying the NIR laser at 1.5 W/cm^2^ for 5 min, the cell viability of ALTS1C1 cells diminished with increasing nanoparticle concentration. Notably, rGOQD/R8/aPDL1 demonstrated greater efficacy in reducing cancer cell viability compared to the rGOQD/R8, which aligns well with the enhanced intracellular uptake efficiency of rGOQD/R8/aPDL1 by ALTS1C1 cells shown in Figure 4d. To confirm the enhanced cytotoxicity from rGOQD/R8/aPDL1, Live/Dead cell staining was carried out and visualized by fluorescence microscopy (Figure 5b). Noticeable PI-stained cells due to late apoptotic and necrotic cell death were only observed in the laser-irradiated groups (rGOQD/R8+L and rGOQD/R8/aPDL1+L) and not the group without laser irradiation ((rGOQD/R8). Furthermore, within the laser groups, fewer dead cells stained with PI and more live cells stained with Calcein-AM were shown for the rGOQD/R8+L group than the rGOQD/R8/aPDL1+L group without aPD-LI-mediated active targeting in rGOQD/R8.

For antigen release from dead cells post photothermal treatment, the CSLM analysis of ALTS1C1 cells treated with rGOQD/R8 or rGOQD/R8/aPDL1 and irradiated with 808 nm laser reveals expression of calreticulin (CRT) on the cell surface with distinctive feature (Figure 5c). Notably, rGOQD/R8/aPDL1 induced a more pronounced CRT expression compared to rGOQD/R8, likely due to a difference in intracellular uptake rates (Figure 5d). Control groups treated with PBS or rGOQD/R8 without laser irradiation show negligible CRT expression, underscoring the role of photothermal response in eliciting DAMP expression. The CRT is one of the significant DAMPs that can be exposed on the surface of dying tumor cells, signaling antigen-presenting cells (APCs) for immune activation [54]. These proteins then trigger pathways that activate immunogenic cell death (ICD) [55]. Antigen presentation, a critical precursor to DC cell activation, is often mediated by the expression of CRT, which is expected when ALTS1C1 cells are experiencing PTT to heighten the immunogenicity of tumor cells and potentially boost the anti-tumor activity [56]. Taken together, our findings suggest that rGOQD-mediated PTT can effectively induce DAMP expression, thereby enhancing the immunogenicity of cancer cells. Such a mechanism may support the use of rGOQD/R8 for tumor photo-immunotherapy.

### 3.3. In Vivo Studies

**Figure 6:**
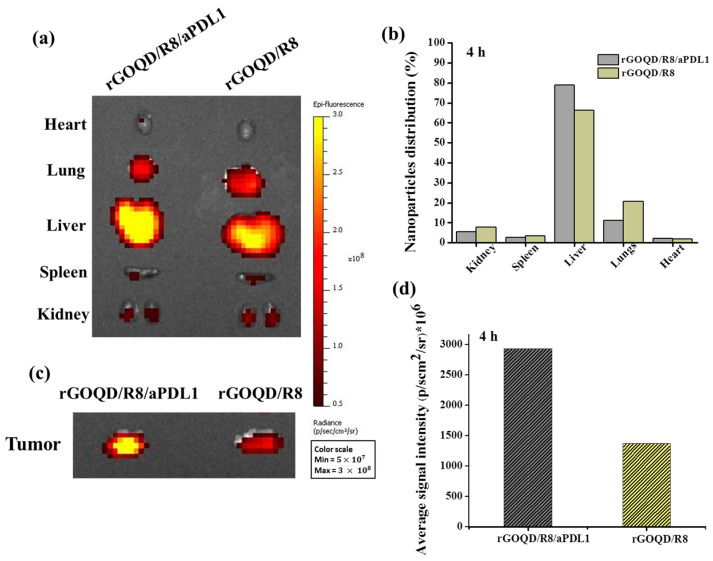
The ex vivo fluorescence images of major organs (**a**) and the corresponding quantification of nanoparticle distribution in each organ from fluorescence intensity (**b**) with an in vivo imaging system (IVIS) 4 h after administration of Cy5.5-labelled rGOQD/R8/aPDL1 or rGOQD/R8 to ALTS1C1 tumor-bearing mice through the tail vein. The ex vivo fluorescence images (**c**) and the corresponding fluorescence intensity (**d**) of tumors with an IVIS 4 h after administration of Cy5.5-labelled rGOQD/R8/aPDL1 or rGOQD/R8 to ALTS1C1 tumor-bearing mice through the tail vein.

The biodistribution and tumor-targeting ability of rGOQD/R8 and rGOQD/R8/aPDL1 were determined in the subcutaneous GBM tumor model using ALTS1C1 cells. The biodistribution study was conducted on six-week-old C57BL/6J mice where Cy5.5-tagged nanoparticles were intravenously injected through the tail vein of tumor-bearing mice. After 4 h, the fluorescence signal from Cy5.5 was detected for excised major organs and tumors ex vivo using an IVIS (Figure 6a). It shows that rGOQD/R8 and rGOQD/R8/aPDL1 were predominantly accumulated in the liver with slight differences between groups, giving clear evidence of hepatic clearance of the nanoparticles via excretion after tail vein injection (Figure 6b). At the same time, the lower fluorescence intensity shown in kidneys indicates a lower rate of renal clearance [57]. Nanoparticles can easily penetrate the tumor region by leaky vasculature and rGOQD/R8 and rGOQD/R8/aPDL1 can be accumulated in the tumors within 4 h (Figure 6c). However, the rGOQD/R8/aPDL1 shows higher fluorescence intensity compared to rGOQD/R8 (2938 × 10^6^ vs. 1379 × 10^6^), due to the active targeting ability of aPD-L1 for PD-L1 on tumor cell surface. Indeed, rGOQD/R8/aPDL1 reveals 2.1 times higher fluorescence intensity than rGOQD/R8 via PD-L1-mediated active targeting (Figure 6d).

**Figure 7:**
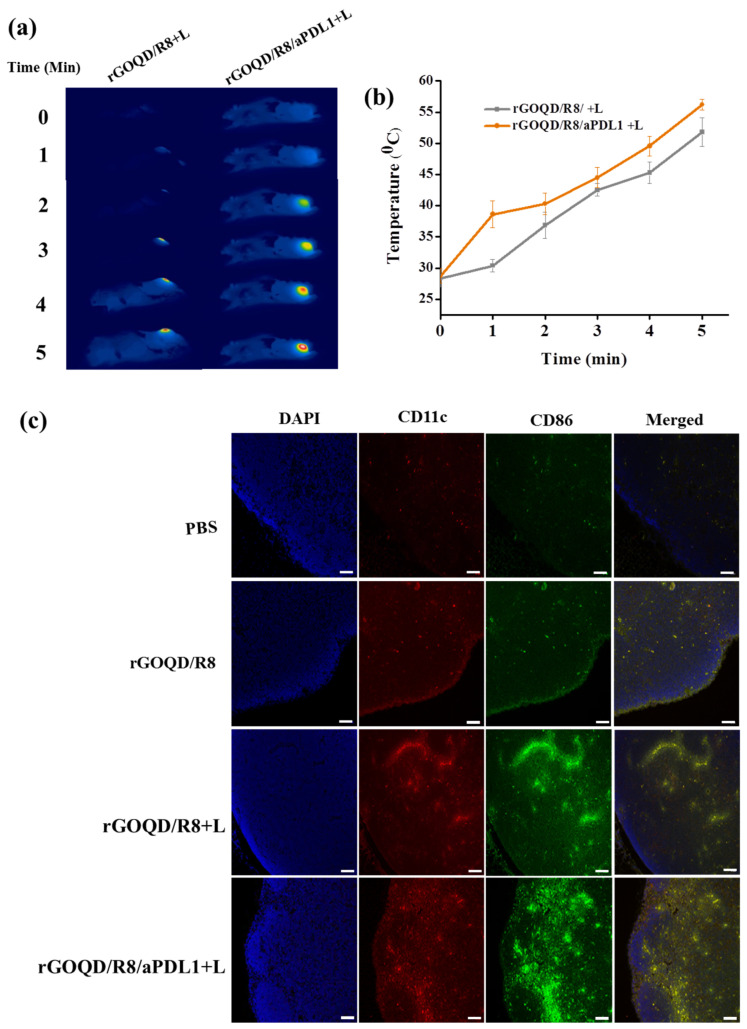
The thermal images (**a**) and the corresponding in vivo peak temperature profiles (**b**) of ALTS1C1 tumor-bearing mice after intravenous injection of rGOQD/R8/aPDL1 or rGOQD/R8 followed by 808 nm NIR laser irradiation 24 h post injection (mean ± SD, *n* = 3). The activation of dendritic cells by rGOQD/R8/aPDL1 or rGOQD/R8 was compared with confocal images of lymph nodes 19 days after treatment by immunofluorescence staining of CD11C (antigen-presenting cells) and CD86 (dendritic cells) in red and green, respectively, and counterstaining the nucleus with DAPI in blue (scale bar = 50 μm) (**c**).

The in vivo therapeutic effect from PTT was evaluated from the tumor temperature change during laser irradiation with thermal images. The peak temperature profile following intravenous injection of both nanoparticles leads to some difference in temperature rise profile after laser irradiation (Figure 7a,b). The mice treated with rGOQD/R8 and rGOQD/R8/aPDL1 showed temperature rises from 28 to 52 °C and from 28 to 56 °C, respectively, after laser irradiation for 5 min. This is attributed to the targeting ability of rGOQD/R8/aPDL1 where prominent intracellular uptake has been shown in vitro. The in vivo photothermal response suggests that both nanoparticles can provide PTT to engage with the immune system by activating ICD after laser exposure [36], which is expected to express surface CRT protein on dying cancer cells. It is reported that neuroblastoma cells (Neuro-2a) treated with different temperature windows during PTT show an appreciable activated ICD, followed by tumor regression with temperature elevation up to 65 °C [58]. The mice treated with a temperature window of 50–65 °C have shown good survival benefits as compared to mild PTT (32–45 °C) and elevated PTT (83 °C) [29]. A favorable ICD is expected with the current treatment protocol where the thermal dose for the treatment can be maintained within 50–60 °C.

The rGOQD/R8/aPDL1 can release R848 after intracellular uptake and released R848 can lead to DC activation. The DC can sense cancer antigens mediated by R848 and convert their immature state to an activated state [6]. The antigen-specific adaptive immune responses can be elicited through the ICD pathway, driving the activation and polarization of APCs loaded with tumor antigen-specific immunotherapy [59]. The antigen-specific immune cell activation can be seen in lymph nodes. The presence of DC in lymph nodes can initiate priming the T-cell activation and infiltration to the tumor [60]. Normally, the immature state of DC will have a lower level of costimulatory molecules such as CD80, CD86, CD83, and MHC II on the cell membrane [61]. When the DC is activated, it can undergo overexpression of the CD86 molecule on its surface, which along with CD11C represents the markers for conventional DC [62]. They are involved in the binding of DC to antigens, facilitating the efficient presentation of antigens to T cells [24]. To evaluate the immune cell activation after combinatory R848 delivery and PTT, mice were sacrificed on the 19th day of treatments. The inguinal lymph nodes were collected and preserved, followed by cutting into 5 μm thickness and co-staining with anti-CD86 and anti-CD11C for the DC activation evaluation (Figure 7c). The mice in the rGOQD/R8, rGOQD/R8+L, and rGOQD/R8/aPDL1+L groups have shown comparatively higher CD11c and CD86 expression than the PBS control group. This is attributed to the release of R848 (TLR7 agonist) in tumor cells by the pH-responsive drug release after endocytosis, thus inducing the antigen presentation and adaptive immune response [36]. Compared to rGOQD/R8, the rGOQD/R8+L and rGOQD/R8/aPDL1+L groups can increase the activated DC expression, due to PTT-mediated ICD activation and immune cell recruitment to the lymph node. A marginal difference between rGOQD/R8+L and rGOQD/R8/aPDL1+L was found from aPDL1-mediated cellular targeting. Overall, these findings suggest that a combination of R848 and PTT can activate DC and recruit T cells into the cancer region for combined photothermal/immunotherapy.

**Figure 8:**
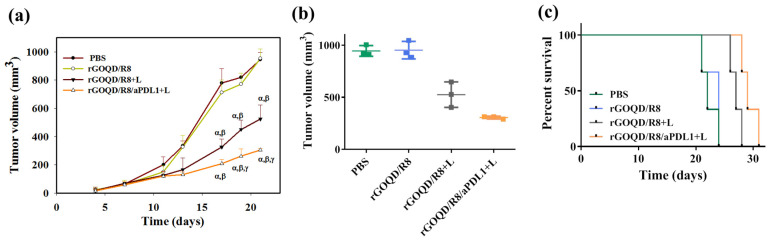
The in vivo therapeutic evaluation was studied using a syngeneic mouse model of GBM with subcutaneously implanted ALTSC1 cells. The mice were divided into four groups and the treatment was initiated by injection of samples on day 10, followed by intravenous injection on days 13, 17, and 20. The control group is PBS and the rGOQD/R8+L and rGOQD/R8/PDL1 groups are with 808 nm NIR laser irradiation at 1.5 W/cm^2^ for 5 min. The tumor volume change (**a**), the scattered plot of tumor volume on day 21 (**b**), and the survival curve of animals (**c**) of ALTSC1 tumor-bearing mice after different treatments (mean ± SD, *n* = 3). The sacrificing criteria were when the tumor volume exceeded 1000 mm^3^. ^α^ *p* < 0.05 compared to PBS, ^β^ *p* < 0.05 compared to rGOQD/R8, ^γ^ *p* < 0.05 compared to rGOQD/R8+L.

The anti-tumor study with the subcutaneous GBM tumor model was evaluated from the change in tumor size over the observation period (Figure 8a). On day 10, after the first treatment, a reduction of the tumor volume was noted for the rGOQD/R8+L and rGOQD/R8/aPDL1+L groups due to imminent photothermal-induced cell death. At the same time, a gradual increase in tumor volume was observed for the PBS and rGOQD/R8 groups without laser irradiation. After the second treatment on day 14, a slower tumor volume increase is noted for the rGOQD/R8/aPDL1+L group than the rGOQD/R8+L group, due to the targeting toward the PD-L1 receptor on the cancer cell surface, which can also enhance the cold tumor nature of GBM towards the hot by recruiting more T cells towards the tumor. A similar trend was observed through day 18 after the third treatment where a significant difference in tumor size starts to show between rGOQD/R8/aPDL1+L and rGOQD/R8+L groups. After the final 4th treatment, the significant difference in tumor volume still exists at day 21, with the mean tumor volume for the PBS group (944 mm^3^) being almost three times that of the rGOQD/R8/aPDL1+L group (306 mm^3^) (Figure 8b). There was a significant tumor size reduction from the rGOQD/R8 group without laser irradiation group to the rGOQD/R8+L group with laser irradiation, due to the combinational effect of immune cell activation by PTT and cancer cell death enhancement through suppressing the PD-L1 and enhancing T-cell recruitment. A survival curve of mice was constructed by setting 1000 mm^3^ tumor volume as the sacrificing criteria (Figure 8c). The mice in the rGOQD/R8/aPDL1+L group show prolonged median survival time (29 days) over mice in both rGOQD/R8 (24 days) and rGOQD/R8+L (27 days) groups, whereas the PBS group show the shortest median survival time of 22 days (Table 2). A statistical analysis of survival times between groups shows the trend in the order of rGOQD/R8/aPDL1+L > rGOQD/R8+L > rGOQD/R8 > PBS. The results manifest the prominent therapy efficacy from photothermal-activated immune cell recruitment, and T-cell recognition of GBM cancer cells by suppressing PD-L1 on the cancer cell surface as well as R848-activated enhanced adaptive immunity.

**Figure 9:**
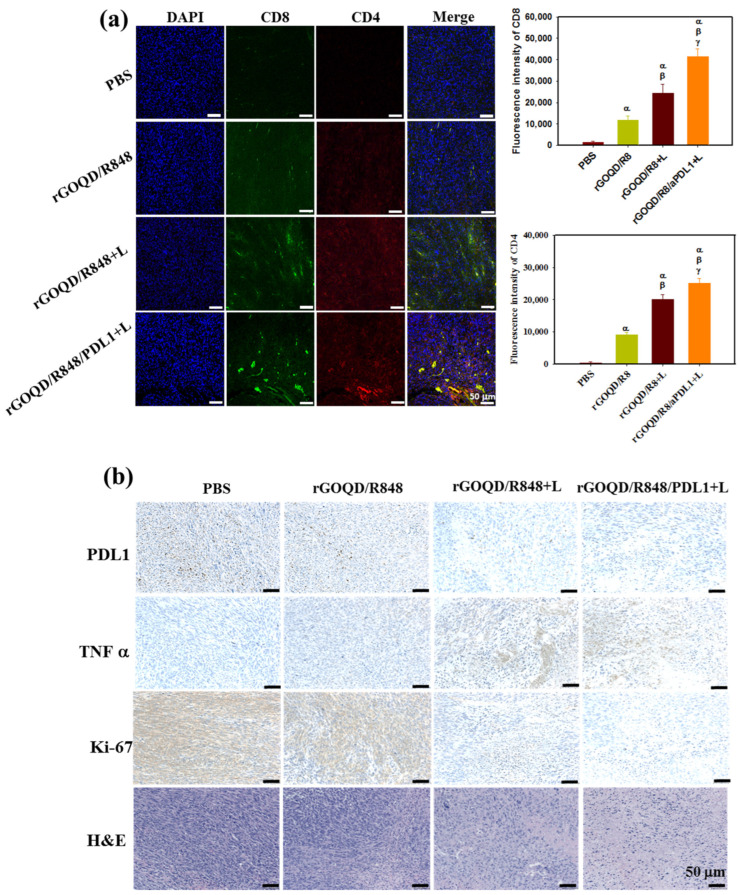
(**a**) In vivo immune response after treatment with rGOQD/R8, rGOQD/R8+L, and rGOQD/R8/aPDL1+L. The infiltration of T cells into the tumor after various treatments was measured by immunofluorescence staining with anti-CD4 and anti-CD8 antibodies. The confocal immunofluorescence images and the corresponding quantification of fluorescence intensity of CD4 and CD8 in the tumor tissues 19 days after treatments (scale bar = 50 μm). (**b**) The immunohistochemistry (IHC) of PD-L1, tumor necrosis factor (TNF-α), and Ki-67, and H&E staining of tumor tissues 19 days after treatments. ^α^ *p* < 0.05 compared to PBS, ^β^ *p* < 0.05 compared to rGOQD/R8, ^γ^ *p* < 0.05 compared to rGOQD/R8+L.

As the observed therapeutic efficacy stems from immune stimulation to provoke an anti-tumor response, the recruitment of cytotoxic T lymphocytes to the tumor milieu was quantitatively assessed 19 days post treatment. The immunohistochemical analysis, depicted in Figure 9a, demonstrated that the rGOQD/R8+L treatment can significantly boost the expression of CD4 and CD8 in the tumor region for photothermal immunotherapy. Furthermore, the rGOQD/R8/aPDL1+L treatment further significantly upregulates the recruitment of CD4^+^ and CD8^+^ T cells to the tumor site and elicits a pronounced augmentation in T-cell infiltration. This can be attributed to the inhibition of PD-L1 interactions on glioblastoma cells with PD-1, thereby potentiating T-cell-mediated cytotoxicity. This mechanism dovetailed with the observed enhancement in survival rates in the murine model, particularly in the rGOQD/R8/aPDL1+L group when combined with PTT. Furthermore, a discernible escalation in CD4^+^ T-cell penetration in the rGOQD/R8+L group relative to the rGOQD/R8 group is indicative of the potentiation of immune response via PTT-induced ICD, which ostensibly recruits an amplified population of anti-tumor immune cells to the tumor site [63]. Conversely, the rGOQD/R8 group was indicative of immune cell recruitment driven by TLR7 activation, which, while effective, cannot match the immune response levels of the combined photo-immunotherapeutic regimens. These findings together with the maturation of DC in the lymph nodes, as shown in Figure 7c, align well with the postulated mechanism of action, by reinforcing the synergistic effect of TLR7 antagonist-mediated T-cell activation in the tumor site with released R848. These results also underscore the potential of integrating aPD-L1 blockade with R848 and PTT, yielding a concerted anti-tumor response as evidenced by the substantial tumor regression in the rGOQD/R8/aPDL1+L group. Therefore, this study not only elucidates the immune cell dynamics in situ but also proposes a viable strategy for enhancing the immunogenicity of GBM cells, thereby improving the prognostic outcomes through innovative drug delivery systems.

In addition to the T-cell recruitment findings, the anti-tumor activity was further assessed through histological analysis of tumor tissue sections. Figure 9b illustrates a marked expression of PD-L1 in the control group, which received PBS. In contrast, the application of rGOQD/R8/aPDL1 resulted in a notable reduction in PD-L1 expression, which can actively target and diminish PD-L1 expression in glioma cells [7]. This effect was mirrored by the effective reduction in tumor volume observed in the rGOQD/R8/aPDL1+L group. The IHC analysis also revealed an upregulation of a key pro-inflammatory cytokine TNF-α in the laser-treated (+L) groups, which is instrumental in PTT-mediated immune cell activation [64]. Particularly, the rGOQD/R8/aPDL1+L group exhibited an increase in TNF-α expression, substantiating the initiation of photo-immunotherapy. Further investigation involving the IHC of Ki-67 protein, a marker for cell proliferation, indicated a high proliferation rate of tumor cells in the PBS control group. This proliferation was markedly reduced in the rGOQD/R8+L and rGOQD/R8/aPDL1 groups, highlighting the therapeutic impact of the treatments. Further confirmation of the tumor proliferation observed from H&E also demonstrated a similar trend with Ki-67. In conclusion, the collective data suggest that the combination of immunotherapy with PTT can effectively modify the tumor microenvironment. This is achieved by activating DC and facilitating the recruitment of T cells, which in turn can exert potent anti-tumor activity. These findings not only offer insights into the mechanisms underlying the anti-tumor effects of rGOQD/R8/aPDL1 but also affirm the potential of nanoparticle-based delivery systems in enhancing immunotherapeutic strategies against GBM.

The safety profile of our treatment regimen was meticulously assessed in tumor-bearing mice, by examining changes in body weight and conducting blood analysis. There were no significant changes in animal body weight among different treatments, which also showed no significant difference from the control group (Figure S7a, Supplementary Materials). This suggests that the nanoparticles and the laser treatment did not adversely affect the overall health of the animals. In addition to body weight, the histopathological examination of vital organs was carried out by H&E staining. Examination of the liver, kidney, lungs, spleen, and heart revealed no noticeable change in cell morphology or minimum tissue damage, thus confirming the biosafety profile of the nanoparticles used in this study (Figure S7b, Supplementary Materials). The hematological analysis of blood samples from sacrificed animals further reinforced the safety (Table S1, Supplementary Materials). No significant differences were observed in blood counts in the treatment groups when compared to the control group. Overall, the rGOQD-based nanoparticles exhibit pronounced safety during the course of the anti-cancer study in mice subcutaneous GBM model, without eliciting detectable systemic toxicity or organ damage. These findings provide a strong foundation for the potential translation of this nanoparticle-based therapy into clinical settings, given the paramount importance of biosafety in the advancement of new therapeutic agents.

## 4. Conclusions

We successfully developed a nanosystem for photothermal immunotherapy of GBM by combining nanoparticle-based R848 immunoadjuvant delivery and PTT with aPD-L1-conjugated rGOQD as a nanovehicle. The conjugation of aPD-L1 to rGOQD can not only actively target the glioma cells but also amplify the immune system’s response against the GBM tumor model. Through the photothermal characteristics of rGOQD upon NIR laser irradiation, PTT can induce the death of cancer cells and lead to the release of DAMPs. This process can further promote the maturation of DC, which is recruited in an immature state upon the release of tumor-specific antigens. The rGOQD/R8/aPDL1 nanoparticle demonstrated significant survival benefits due to the recruitment of cytotoxic T lymphocytes within the tumor region. Moreover, its biocompatibility and non-toxic nature underscore a broader application in cancer combination therapy. From the in vivo study with a subcutaneous GBM tumor model, intravenous delivery of rGOQD/R8/aPDL1 can target the tumor and provide promising anti-tumor effects upon NIR laser irradiation. Overall, this combinational GBM therapeutic approach can potentially transform the tumor microenvironment into one that is responsive to immune interventions by leveraging PTT and immunotherapy in a single nanoplatform to advance brain tumor therapy.

## Figures and Tables

**Table 1:** The average particle size and polydispersity index (PDI) from dynamic light scattering analysis and the average zeta potentials.

Particles	Size (nm)	PDI	Zeta Potentials (mV)
GOQD	17 ± 4	0.19 ± 0.11	−54.2 ± 7.0
rGOQD	79 ± 8	0.13 ± 0.05	43.2 ± 1.5
rGOQD/R8	120 ± 14	0.20 ± 0.14	32.3 ± 2.1
rGOQD/R8/aPDL1	150 ± 7	0.22 ± 0.08	25.6 ± 1.0

**Table 2:** The survival analysis of ALTS1C1 tumor-bearing mice after different treatments.

Group	Median Survival Time (Days)	Average Survival Time ^1^
PBS	22	22.3 ± 1.3
rGOQD/R8	24	23.0 ± 1.4
rGOQD/R8+L	27	27.0 ± 0.8 ^α,β^
rGOQD/R8/aPDL1+L	29	29.0 ± 0.8 ^α,β,γ^

^1^ Mean ± standard deviation (SD). ^α^ *p* < 0.05 compared to PBS, ^β^ *p* < 0.05 compared to rGOQD/R8, ^γ^ *p* < 0.05 compared to rGOQD/R8+L.

## Data Availability

The data presented in this study are available on request from the corresponding author.

## Supplementary Materials

The following are available online at https://www.mdpi.com/article/10.3390/pharmaceutics16081064/s1, Figure S1: The TEM image of rGOQD; Figure S2: The UV-Vis spectrum of R848; Figure S3: The Raman spectroscopy, XRD, XPS, and AFM analysis of rGOQD/R8; Figure S4: The photothermal stability and the effects of different laser power intensities; Figure S5: The calibration curve of R848 and the loading of R8484 to rGOQD; Figure S6: The gross observation of stability of rGOQD/R8/aPDL1 in pH 7.4 PBS and DMEM; Figure S7: The change in animal body weight and the H&E staining images of major organs retrieved from sacrificed animals; Table S1: The blood analysis for evaluation of systemic toxicity.
