# Supplementary material for: Immunomodulatory R848-Loaded Anti-PD-L1-Conjugated Reduced Graphene Oxide Quantum Dots for Photothermal Immunotherapy of Glioblastoma
Source: Pharmaceutics. 2024 Aug 13;16(8):1064. doi: 10.3390/pharmaceutics16081064 (PMC11358977; doi:10.3390/pharmaceutics16081064)
Supplement: Supplementary file 1 [file pharmaceutics-16-01064-s001.zip › pharmaceutics-3102673-supplementary.pdf]

Supplementary Materials

Immunomodulatory R848-Loaded Anti-PD-L1-Conjugated Reduced Graphene Oxide Quantum Dots for Photothermal Immunotherapy of Glioblastoma

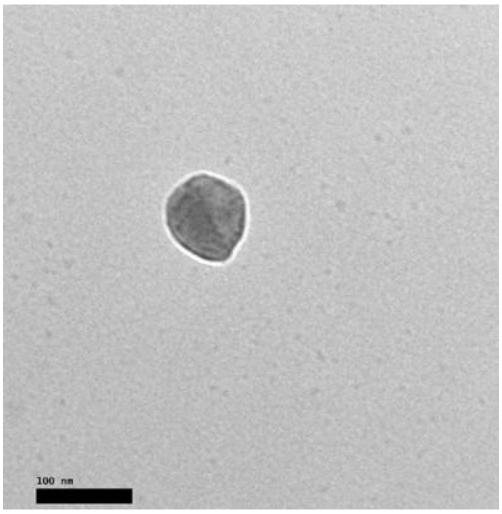

Figure S1. The transmission electron microscope (TEM) image of rGOQD. Bar = 100 nm.

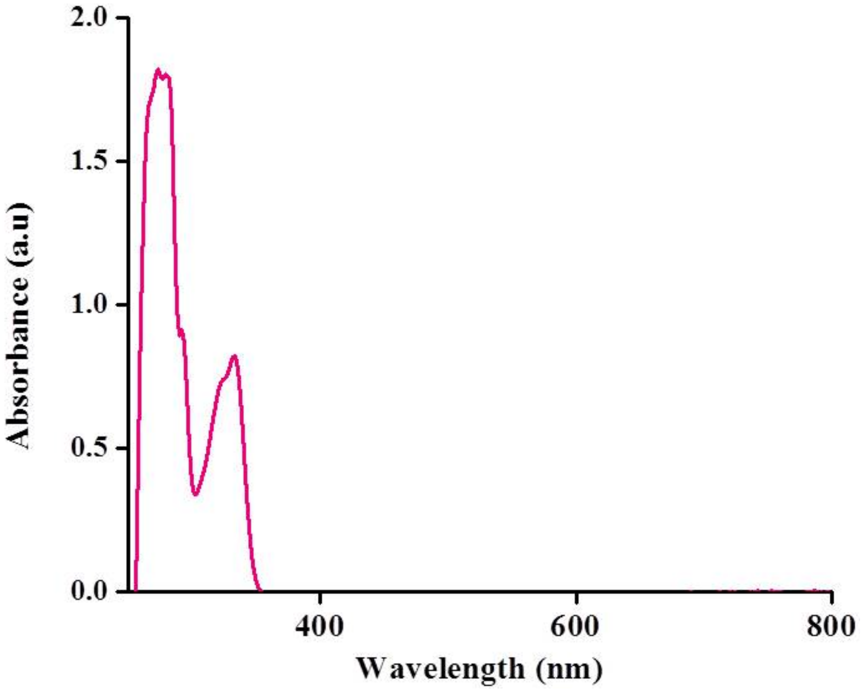

Figure S2. The UV-Vis spectra of R848.

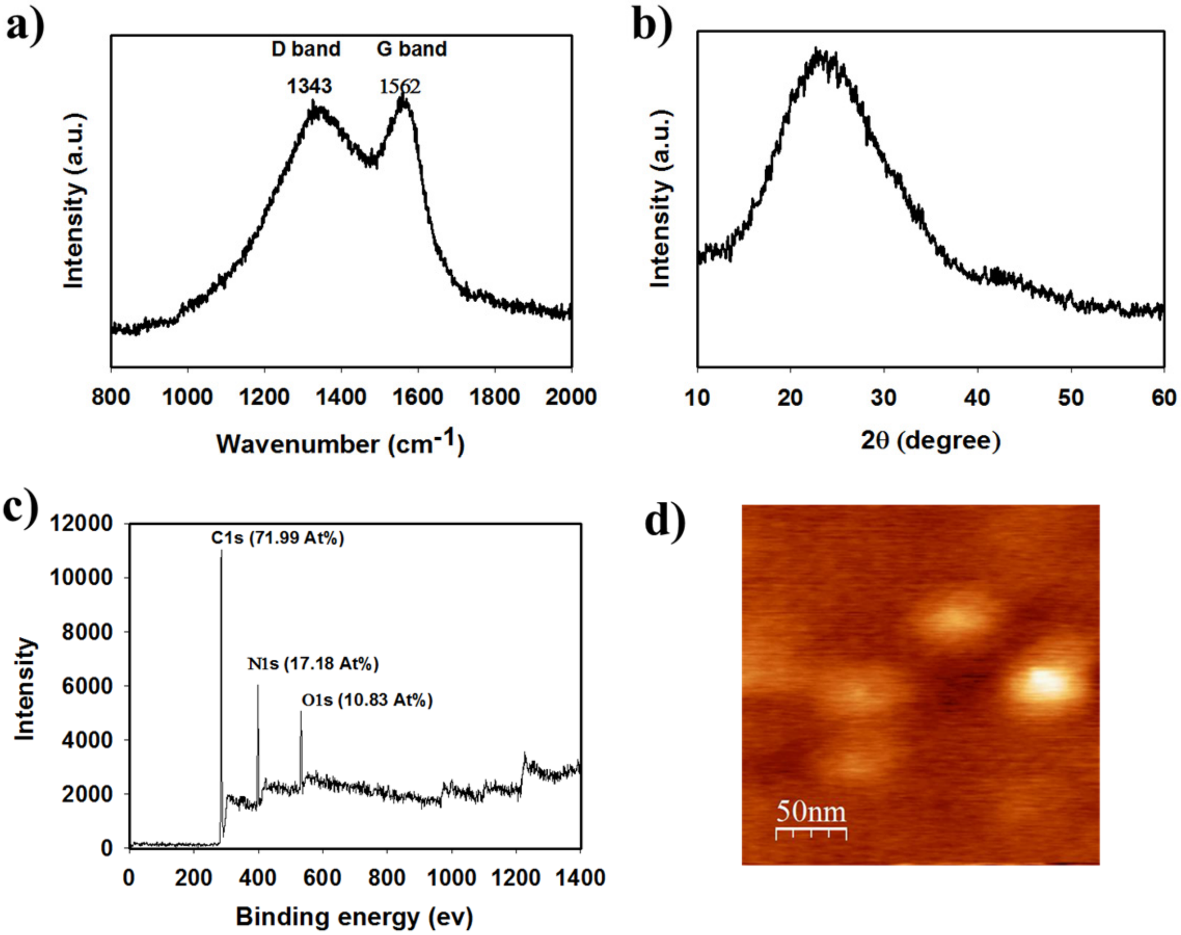

Figure S3. The Raman spectroscopy (**a**), X-ray diffraction (XRD) (**b**), X-ray photoelectron spectroscopy (XPS) (**c**), and atomic force microscopy (AFM) (**d**) analysis of rGOQD/R8.

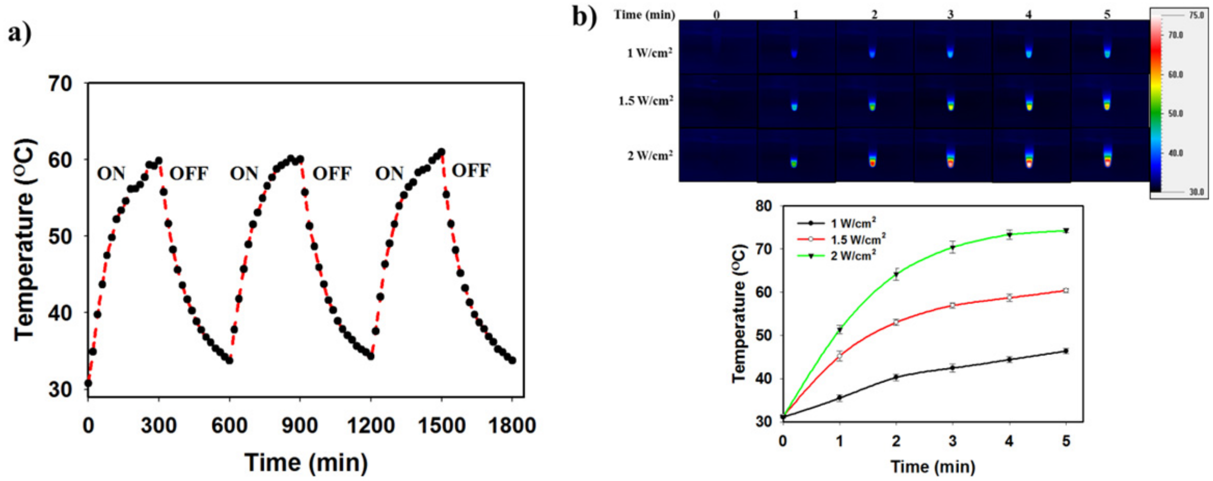

Figure S4. (**a**) The photothermal stability by irradiating rGOQD/R8 (100 μg/mL) in 3 consecutive on/off cycles with 808 nm laser (1.5 W/cm^2, 5 min). (**b**) The photothermal images and the corresponding temperature profiles by irradiating rGOQD/R8 (100 μg/mL) with 808 nm laser for 5 min at different laser power intensities.

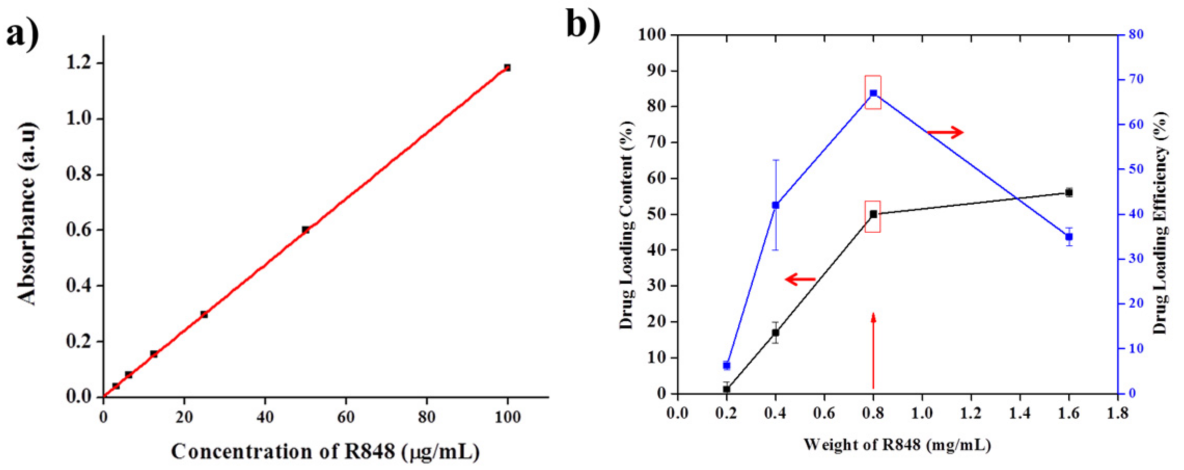

Figure S5. The calibration curve of R848 (**a**), and the loading of R8484 to rGOQD (**b**).

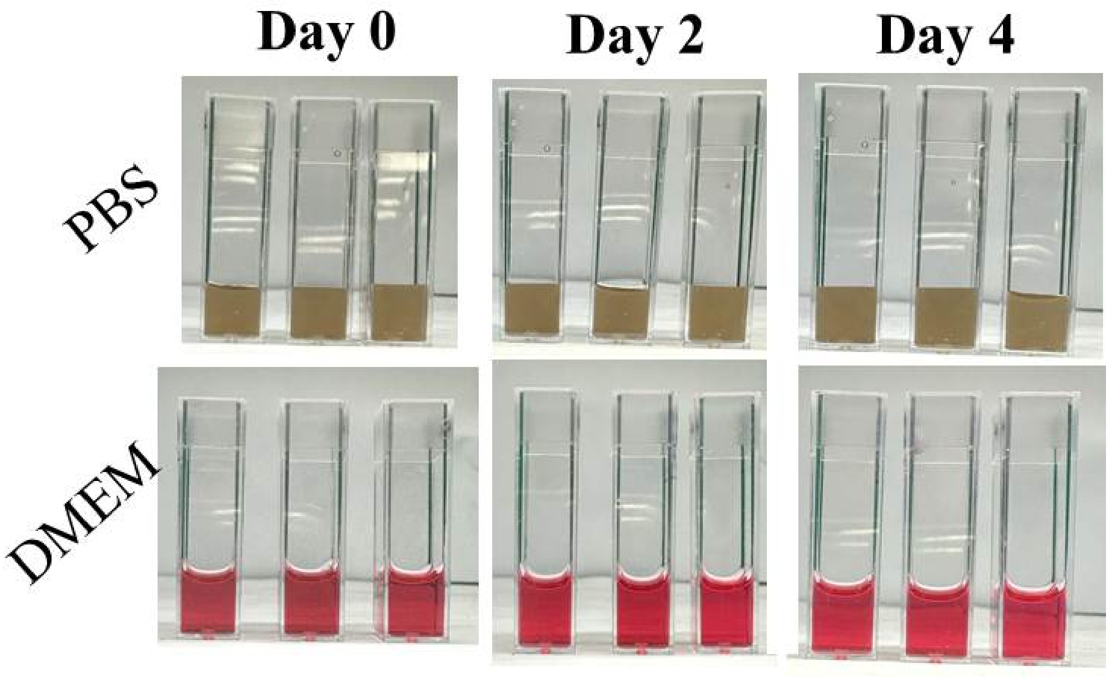

Figure S6. The gross observation of stability of rGOQD/R8/aPDL1 in pH 7.4 PBS and DMEM (containing 10% FBS).

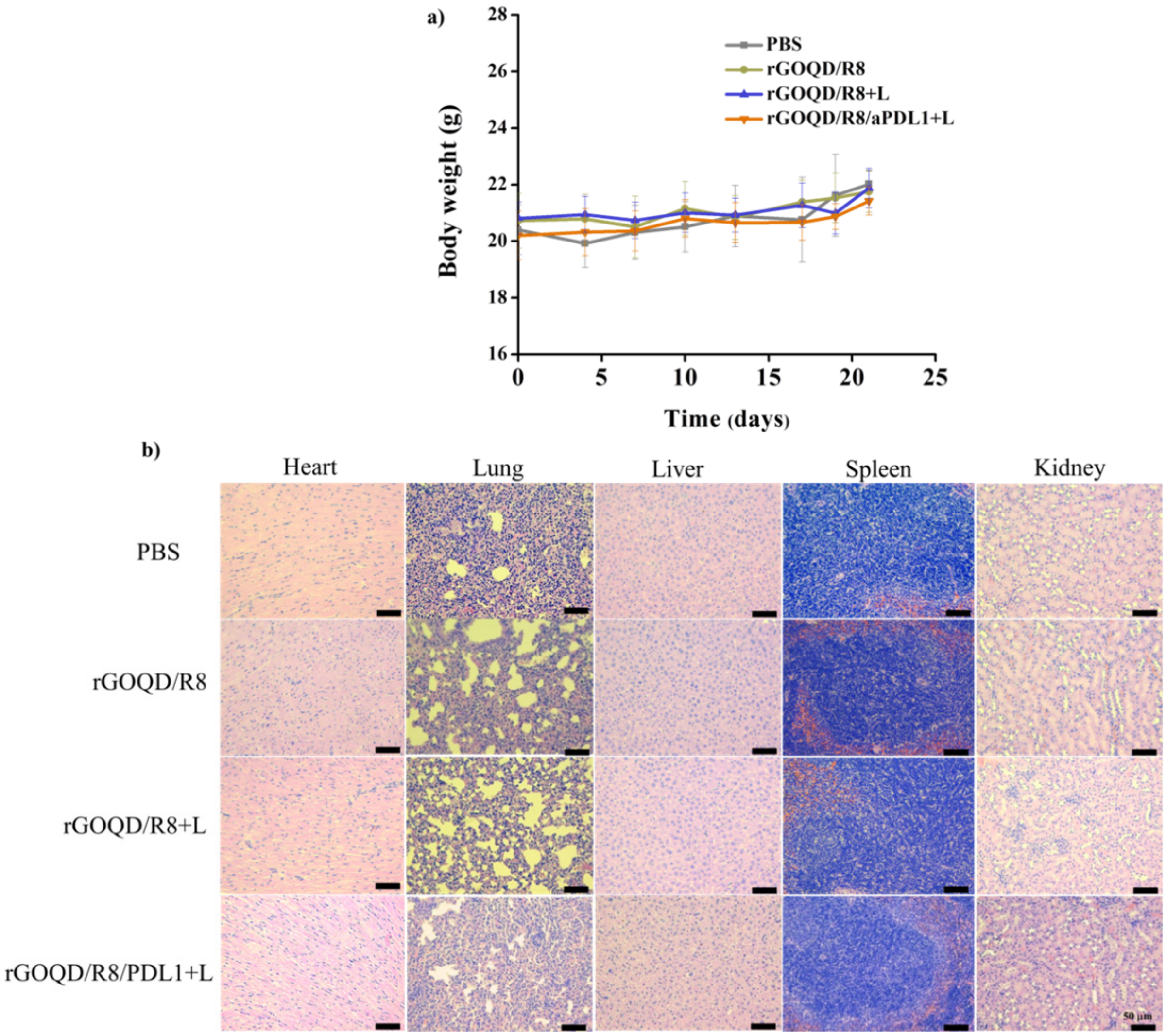

Figure S7. The change in animal body weight up to 21 days (a) and the H&E staining images of major organs retrieved from sacrificed animals on day 19 (b). Bar = 50 μm.

Table S1. The blood analysis for evaluation of systemic toxicity (n = 3, mean ± SD)

Item	Unit	PBS	rGOQD/R8	rGOQD/R8+L	rGOQD/R8/aPDL1+L
**WBC**	10^3 cells/µL	3.6 ± 0.92	2.9 ± 0.95	3.6 ± 1.16	3.4 ± 1.39
**RBC**	10^6 cells/µL	8.5 ± 0.74	9.0 ± 0.48	9.6 ± 0.36	8.9 ± 0.11
**HGB**	g/dL	12.5 ± 1.18	13.1 ± 0.71	13.8 ± 0.35	13.0 ± 0.30
**HCT**	%	46.1 ± 3.75	48.5 ± 2.97	51.0 ± 1.29	48.0 ± 1.70
**PLT**	10^3 cells/µL	344.3 ± 82.3	323.5 ± 37.5	503.0 ± 107.6	433.3 ± 127.2

WBC: white blood cell, RBC: red blood cell, HGB: hemoglobin, HCT: hematocrit, PLT: platelets.
